# Temporal dynamics of walnut phyllosphere microbiota under synergistic pathogen exposure and environmental perturbation

**DOI:** 10.3389/fmicb.2025.1551476

**Published:** 2025-04-01

**Authors:** Shiwei Wang, Yu Tan, Qing Luo, Xinmei Fang, Hanmingyue Zhu, Shuying Li, Yujue Zhou, Tianhui Zhu

**Affiliations:** ^1^College of Forestry, Sichuan Agricultural University, Chengdu, Sichuan Province, China; ^2^Chengdu Botanical Garden, Chengdu, Sichuan Province, China; ^3^Life Science College, Neijiang Normal University, Neijiang, China; ^4^College of Landscape Architecture, Sichuan Agricultural University, Chengdu, Sichuan Province, China; ^5^National Forestry and Grassland Administration Key Laboratory of Forest Resources Conservation and Ecological Safety on the Upper Reaches of the Yangtze River, Chengdu, Sichuan Province, China

**Keywords:** plant-microbe interactions, phyllosphere microbiota, leaf disease, *Juglans regia* L., high-throughput sequencing

## Abstract

**Introduction:**

Phyllosphere-associated microbes directly influence plant-pathogen interactions, and the external environment and the plant shape the phyllosphere microbiome.

**Methods:**

In this study, we integrated 16S rRNA and ITS high-throughput sequencing to systematically investigate changes in the phyllosphere microbiome between symptomatic and asymptomatic walnut leaves affected by spot disease, with consideration of phenological stage progression. Additionally, we explored how abiotic (AT, DT, SCTCC & LPDD) and biotic factors (*Pn* & *Gs*) impact microbial communities.

**Results:**

Our findings revealed significant differences in the diversity of the phyllosphere microbiome between symptomatic and asymptomatic leaves at the same phenological stage. Furthermore, the structure and function of phyllosphere-associated microbiome changed as the phenological stage progressed. Fungal taxa that related to the function Plant_Pathogen and bacterial taxa that related to the KEGG pathway functions Fatty acid biosynthesis and Biotin metabolism were increased in the symptomatic group. The keystone species driving the walnut phyllosphere microbiome was *Pseudomonas* spp., which substantially influenced the microbiome of symptomatic vs. asymptomatic leaves. Notably, *Pseudomonas* spp. interacted with *Xanthomonas* spp. and *Pantoea* spp. Correlation analysis revealed that the dew point temperature constituted the primary abiotic factor of phyllosphere bacterial community composition, whereas liquid precipitation depth dimension was identified as the dominant factor shaping fungal taxa. Additionally, leaf net photosynthetic rate and stomatal conductance were closely linked to the phyllosphere microbiome.

**Discussion:**

These results advance our understanding of community-level microbial responses to pathogen invasion and highlight the multifactorial drivers of phyllosphere microbiome assembly. Ultimately, they contribute to predicting and managing walnut leaf-related diseases.

## 1 Introduction

In nature, plants and microorganisms form an intricate network. They can colonize the surface or interior of plants and be classified as rhizosphere microorganisms, phyllosphere microorganisms, seed microorganisms, and more, depending on the location of their habitats. Research on the plant microbiome has historically focused on the soil and rhizosphere microbiomes, with nearly three times more attention than on the phyllosphere microbiome (Koskella, 2020). The phyllosphere microbiome primarily resides in the above-ground part of the plant, and the foliar microbiome alone can contain up to 10^26^ bacterial cells, originating from sources such as air, rain, and insects (Peñuelas and Jaume, 2014). Additionally, soil microorganisms can be transferred to the phyllosphere through the xylem and phloem (Bell et al., 2021). Abiotic factors—such as temperature, humidity, light, and nutrients—influence the phyllosphere microbial community, while biotic factors such as plant species, age, and condition also play a role (Laforest-Lapointe et al., 2016; Li et al., 2021; Alsanius et al., 2017; Thapa et al., 2017; Chen et al., 2021). Plant-microbe-environment interactions become more crucial given the growing impact of human activities and environmental degradation on plant ecosystems in recent years. Like rhizosphere microorganisms, phyllosphere microorganisms are crucial for maintaining host homeostasis and enhancing host defense through microbial interactions, host metabolism regulation, and immune system triggering (De Mandal and Jeon, 2023). The phyllosphere microbiome experiences changes in diversity and abundance under biotic or abiotic stresses, influenced by microbial interactions involving keystone taxa—key regulators that shape community structure (Agler et al., 2016). The host and microbiome engage in two-way cooperative strategies, such as biomass production and nitrogen fixation, to boost host growth and facilitate plant recruitment of microorganisms in the phyllosphere for pathogen defense (Abadi et al., 2020; Li et al., 2022). The phyllosphere microbiome can be influenced by plant leaf infections, often manifesting as a reduction in diversity and a shift in dominant communities, and it has been demonstrated in studies on soybean, tomato, and *Arabidopsis thaliana* that these changes play a crucial role in maintaining plant health (Chen et al., 2020; Díaz-Cruz and Cassone, 2022; Ehau-Taumaunu and Hockett, 2023; Goossens et al., 2023). To gain a deeper understanding of plant-microbe, microbe-microbe, and plant-microbe-environment interactions, exploring the relationship between pathogen invasion and the phyllosphere microbiome at the community level is essential.

Walnut (*Juglans regia* L.) leaf spotting diseases, including black spot, brown spot, and wilt, occur worldwide and can induce a wide range of destructive symptoms. These symptoms range from damage to leaves, branches, and fruits, severely jeopardizing their growth and yield. Interestingly, walnut pathogens that cause foliar diseases show multi-pathogen complex infestations or cross-over. Specifically, *Xanthomonas campestris* pv. juglandis is the primary causal agent for walnut black spot disease. Additionally, *Pseudomonas syringae, Pantoea agglomerans*, and *Alternaria* spp. serve as companion pathogens (Keshtkar et al., 2016). However, *Pseudomonas flavescens*, *Alternaria* spp., and *Fusarium* spp. are reported as the pathogens responsible for causing walnut blight (Kałużna et al., 2014; Kim et al., 2020), *Fusarium* spp., *Alternaria* spp., *Phomopsis* spp., *Cladosporium* spp., and *Colletotrichum* spp. contribute to brown spot disease in walnuts (Liu et al., 2023). Maybe pathogen crossover partly explains why disease etiology is not always straightforward under natural conditions; rather, disease occurs due to interactions between multiple pathogens. The phyllosphere microbiome may offer some resistance to leaf diseases, and understanding how the phyllosphere microbiome responds to walnut leaf spotting diseases can aid in developing more effective strategies for prevention and control.

In this study, we integrated high-throughput sequencing to investigate and compare the phyllosphere microbiomes between symptomatic (black/brown/any abnormal spots) and asymptomatic leaves from walnut trees under natural field conditions. This study aimed to (i) characterize the composition, diversity, and structural shifts in the phyllosphere microbiome between symptomatic and asymptomatic leaves, (ii) analyze how changes in phyllosphere microbial diversity and community structure affect microbiome functions, and (iii) analyze whether and how the phyllosphere microbiome of symptomatic and asymptomatic leaves is affected by abiotic and biotic factors in different phenological stages. The results provide potential insights for managing leaf diseases in walnuts, which are globally significant nuts.

## 2 Materials and methods

### 2.1 Sample collection and processing

Two groups of leaf samples (symptomatic and asymptomatic) of *Juglans regia* L. (“chuanzao” cultivar) were collected from an orchard in Ma Lie Township, Hanyuan County, Ya’an City, Sichuan Province, China (29°20ʹ N, 102°46ʹ E). The symptomatic and asymptomatic leaves were identified based on visual spotting disease symptoms (black/brown/any abnormal spots disease area) and the degree of disease (Supplementary Figure S1; Supplementary Table SI). Five asymptomatic and five symptomatic walnut trees, each of similar size and growth conditions, were selected. Sixty leaves (symptomatic or asymptomatic) per tree were collected; five well-developed and fully mature leaves were selected from each canopy layer (upper, middle, and lower) in every direction (east, west, south, and north). All leaves were carefully placed in sterile Ziploc bags, promptly transported to the laboratory within 12 h, and stored at −80°C. Symptomatic or asymptomatic leaves at each sampling time point were homogeneously mixed evenly, and 50 leaves of similar size from the same tree were chosen as one sample for high-throughput sequencing analysis in this study. The sampling timing was determined based on the phenological stage of walnuts, including the fact that leaves were sampled at five time points across the season, aiming to reflect the dynamics of the microbial community on walnut leaves. Sampling periods included mid-May (leaf expansion period), mid-June (after the leaves were fully expanded), early July to late August (fruit swelling), late August to early September (the core-hardening stage), and early October (before leaves fell) in 2018. Asymptomatic (healthy, he) leaves were marked as HE/he groups, and symptomatic (infected, in) leaves were marked as IN/in groups, upper case letters are fungal samples, and lower case letters are bacterial samples, as shown in Supplementary Table SI.

### 2.2 Isolation of phyllosphere isolates and DNA extraction

The collection of microorganisms on the leaves referenced the methods by Donegan et al. (1991), Rossetti et al. (2017) and Fan et al. (2024), and certain modifications were made. After mixing the 50 walnut leaves, 30 leaves were selected and cut into 5 × 5 mm fragments using scissors (sterilized at 121°C). Subsequently, the pieces were transferred to a sterilized triangular flask, and 200 mL of sterile 0.9% NaCl solution was added. The flask was sealed with a breathable sealing membrane and incubated on a shaker at 4°C, 120 rpm for 1 h. Afterward, the walnut leaf residue was filtered through a layer of sterile gauze, and the resulting filtrate was centrifuged at 14,000 × g, 4°C for 30 min. The precipitate was resuspended in a small sterile 0.9% NaCl solution. This same processing procedure was applied to each sample. Asymptomatic and symptomatic leaves constituted five distinct groups per time point of samples. Total genomic DNA was extracted using the modified cetyltrimethylammonium bromide (CTAB) method (Stewart and Via, 1993).

### 2.3 Illumina HiSeq sequencing of phyllosphere microbes, data processing, and OTU table generation

The primers 515F (5’-GTGCCAGCMGCCGCGG-3′) and 806R (5’-GGACTACHVGGGTWTCTAAT-3′) were used for the amplification of the bacterial 16S rRNA V4 region and the primers ITS5-1737F (5’-GGAAGTAAAAGTCGTAACAAGG-3′) and ITS2-2043R (5’-GCTGCGTTCTTCATCGATGC-3′) were used for the amplification of the fungal ITS1 region. The library was constructed using a TruSeq^®^ DNA PCR-Free Sample Preparation kit. The completed library was quantified by Qubit and Q-PCR and sequenced using a HiSeq 2,500 PE250 platform. Paired-end reads were assigned to samples based on their unique barcode and truncated by cutting off the barcode and primer sequence, then they were merged using FLASH (V1.2.7),^1^ and the splicing sequences were called raw tags. The spliced sequences were filtered to obtain high-quality tags in the effective tags. Sequences with ≥97% similarity were assigned to the same operational taxonomic units (OTUs) by Uparse software (Uparse v7.0.1001).^2^ Species classification was performed with the Mothur algorithm (Schloss et al., 2009) and the SILVA132 SSU rRNA database,^3^ applying a confidence threshold of 0.8–1. This pipeline generated taxonomic information and community composition profiles across all hierarchical levels (kingdom to species).

### 2.4 Phyllosphere microbes diversity, composition and functional predictions analysis

QIIME (Version 1.9.1) was used to analyze the samples’ PD_whole_tree, Shannon, Simpson, Chao1, and ACE diversity index to analyze the alpha diversity of the samples (Caporaso et al., 2010). Bar and Venn diagrams were used to visualize the uniqueness and overlap of OTUs detected in symptomatic versus asymptomatic leaves across five sampling time points. We combined multiple sequence comparisons and relative abundance comparisons to analyze changes in microbial community compositions. Non-metric multi-dimensional scaling (NMDS) and multiple response permutation procedure (MRPP) were conducted at the genus level to investigate differences in microbial community structure between groups. Species differing between groups were further analyzed based on linear discriminant analysis effect size (LEfSe) and similarity percentage (SIMPER) analysis. For network analyses, co-occurrence networks of the samples were performed using the Spearman correlation calculated to assess the complexity of the interactions in phyllosphere microbiota. The function prediction of fungal and bacterial OTUs was performed with the FUNGuild and PICRUSt2.

### 2.5 Identification of correlations between phyllosphere microbes and the environment (AT, DT, SCTCC & LPDD) by RDA/CCA, mantel-test and VPA methods

Air temperature (AT), dew point temperature (DT), sky condition total coverage code (SCTCC), and liquid precipitation depth dimension - six-hour duration (LPDD) with microbial communities were analyzed by canonical correspondence analysis/redundancy analysis (RDA/CCA) analysis, Mantel analysis (Mantel-test), and Spearman correlation calculated. These are used to explain the degree of correlation between environmental factors and community and species distributions. Further, variance partitioning analysis (VPA) was conducted to quantify the amount of species distribution explained by one or more of these factors. Environmental data can be accessed from the National Oceanic and Atmospheric Administration, National Centers for Environmental Information.^4^

### 2.6 Identification of correlations between phyllosphere microbes and plant performance

A Li-6800 portable photosynthesis-fluorescence analyzer (Li-Cor, Lincoln, NE, United States) was used for leaf photosynthetic properties. The flow rate was 500 μmol·s^−1^, the concentration of CO_2_ was 400 μmol·mol^−1^, the photosynthetic active radiation was 800 μmol·m^−2^s^−1^, and the time 9:00–11:30 (at every sampling time point) was when we measured the photosynthetic index. Walnut trees which selected to collect high-throughput sequencing analysis samples as biological replicates for each sampling. Three mature leaves at the middle canopy layer were sampled from each tree, with the most severe infection site as the central measurement point. Net photosynthetic rate (Pn) and stomatal conductance (Gs) measurements were conducted in triplicate and averaged for statistical analysis. Sampling positions on asymptomatic leaves were designated using the corresponding measurement point on symptomatic leaves as central reference points for physiological assessments. The effects of leaf photosynthetic characteristics on the phyllosphere microbial community were analyzed by canonical correspondence analysis/redundancy analysis (RDA/CCA).

### 2.7 Data analysis

The Wilcox test was employed to assess the statistical significance of differences in relative abundance between sample groups, and the Bray-Curtis distance was used to analyze the extent of differences between sample groups and their community distribution patterns. R software (version 3.6.3, the R Foundation for Statistical Computing, Vienna, Austria) was used for NMDS, MRPP, LEfSe, SIMPER, CCA/RDA, Mantel-test, and VPA, even generating figures. Co-occurrence networks were generated by Graphviz (version 2.38.0) and Cytoscape (version 3.9.1). FUNGuild and PICRUSt2 were carried out through the free online platform of Wekemo Bioincloud.^5^

## 3 Results

### 3.1 Shared OTUs of phyllosphere communities in symptomatic and asymptomatic groups

**Figure 1:**
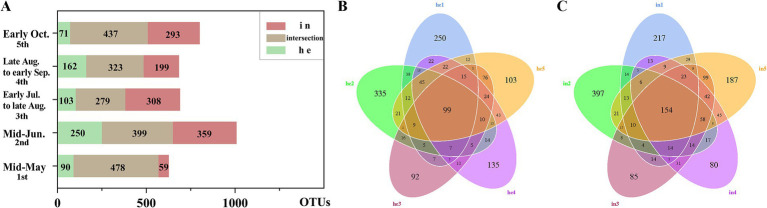
Differences in overall composition with symptomatic and asymptomatic walnut phyllosphere microbial communities at operational taxonomic unit (OTU) level. **(A)** Stacked bar charts: differences in shared and exclusive OTUs of microbial communities in symptomatic and asymptomatic leaves at each sampling time for bacteria; **(B)** Flower plot: differences in the number of OTUs in samples from the asymptomatic group at different sampling times; **(C)** Venn plot: differences in the number of OTUs in samples from the symptomatic group at different sampling times.

In this study, we obtained 75,476 sequences for 16S rRNA amplicon sequencing and 83,312 sequences for ITS1 amplicon sequencing from each group of leaf samples. The bacterial and fungal sequence data were deposited in the NCBI database under the SRA accession of PRJNA873261 and PRJNA600291, respectively. After data analysis, high-quality sequences were clustered into 1,998 16S rRNA and 2,155 ITS operational taxonomic units (OTUs) with 97% identity. Between-group community differences (HE vs. IN) in fungi was published in a separate paper.^6^ Phyllosphere bacterial community composition also differed between the symptomatic and asymptomatic groups. At each sampling time, the percentage of shared OTUs (% = shared OTUs/total OTUs) in the symptomatic and asymptomatic groups each were 76.23%, 39.58%, 40.43%, 47.22 and 54.56% (Figure 1A). Asymptomatic leaves also differed in the community composition of bacteria at different phenological stages of walnut. The proportion of shared OTUs was slightly higher in the symptomatic groups (4.91%) than in the asymptomatic groups (3.82%) throughout the sampling cycle (Figures 1B,C).

### 3.2 Phyllosphere bacterial abundance and diversity in symptomatic and asymptomatic groups

**Figure 2:**
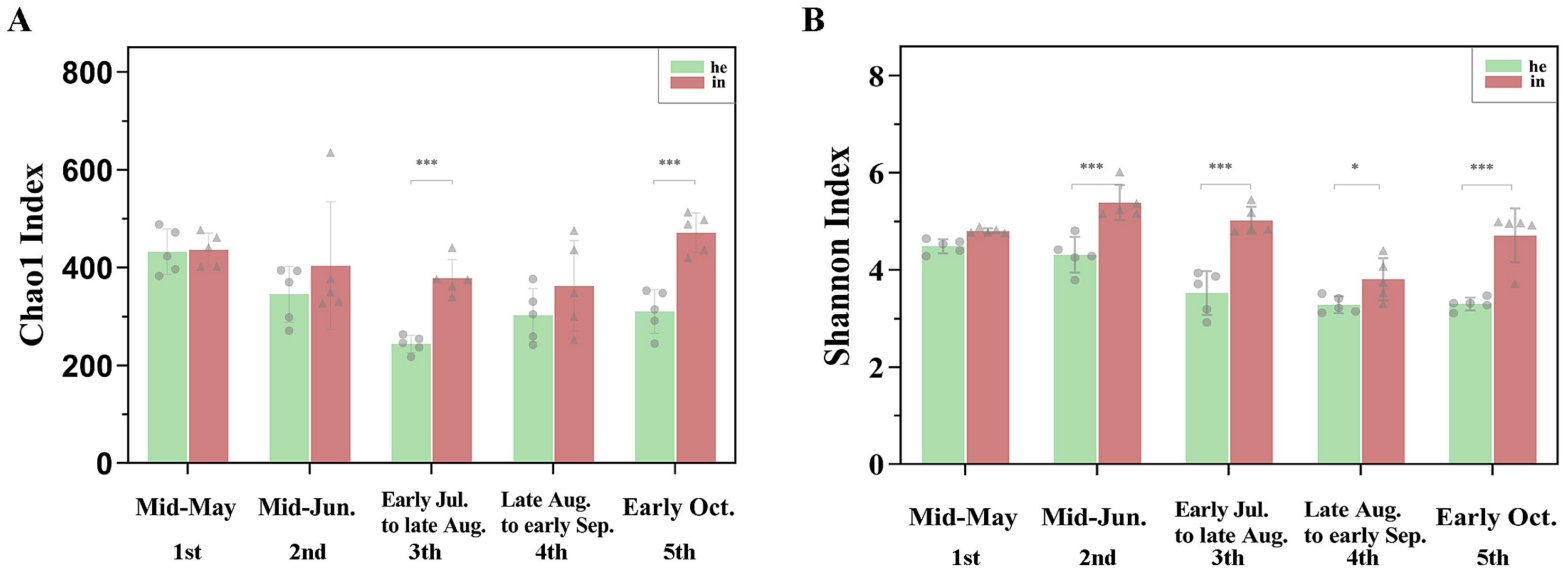
Differences in richness and diversity indices of phyllosphere microbial communities associated with symptomatic and asymptomatic walnut leaf. **(A)** Chao1 index for richness; **(B)** Shannon index for diversity. Different number of * indicate significant differences between symptomatic and asymptomatic walnut leaf, as follows: *0.01 < *p* ≤ 0.05; **0.001 < *p* ≤ 0.01; ***p ≤ 0.001.

Alpha diversity indices include Shannon, Chao1, ACE, PD_whole_tree, and Simpson (Supplementary Table SII). Bacterial community richness and diversity were higher in the symptomatic group compared to the asymptomatic group in the same period. With the development of the phenological period, the phyllosphere bacterial richness index (Chao1) and diversity index (Shannon) changed, and two indices are highly significant differences (*p*-value <0.001) were observed at both the he3 vs. in3 and he5 vs. in5 (Figures 2A,B; Table 1). NMDS at the genus level (Stress <0.2) quantified shifts in walnut phyllosphere microbiota. Peak community distance between symptomatic and asymptomatic leaves occurred at the third sampling. As the phenological period progressed, phyllosphere fungal communities clustered in the fourth and fifth samplings, while bacterial communities remained relatively separated at each sampling, indicating that the phenological period affects bacteria more than fungi (Supplementary Figures S3A,B). Further, the multiple response permutation procedure (MRPP) showed that infection significantly impacted (*p < 0.05*) the diversity of phyllosphere microbial bacterial communities (Table 2).

**Table 1:** The non-parametric Wilcox test results.

	Difference	*p* value	Sig.	LCL	UCL
The non-parametric Wilcox test results of Chao 1 index					
he1-in1	−1.6	0.7914		−13.7466	10.54665
he2-in2	−4.4	0.4684		−16.5466	7.746646
he3-in3	−22.6	5.00E-04	***	−34.7466	−10.4534
he4-in4	−10.2	0.0974	.	−22.3466	1.946646
he5-in5	−29	0	***	−41.1466	−16.8534
The non-parametric Wilcox test results of Shannon index					
he1-in1	−7.2	0.0762	.	−15.1939	0.793858
he2-in2	−21.6	0	***	−29.5939	−13.6061
he3-in3	−28.8	0	***	−36.7939	−20.8061
he4-in4	−9.8	0.0175	*	−17.7939	−1.80614
he5-in5	−28.4	0	***	−36.3939	−20.4061

sig. indicates whether it is significant or not; *p-value < 0.05, **p-value < 0.01, ***p-value < 0.001; LCL, Lower Confidence Limit; UCL, Upper Confidence Limit.

**Table 2:** Multiple response permutation procedure (MRPP) of differences in phyllosphere bacteria structure between symptomatic and asymptomatic group.

Group	A	Observed-delta	Expected-delta	Significance (*p* value)
he1-in1	0.06706	0.28	0.3001	0.011
he2-in2	0.2777	0.3075	0.4258	0.007
he3-in3	0.4039	0.3196	0.5361	0.009
he4-in4	0.6013	0.195	0.4891	0.008
he5-in5	0.4341	0.238	0.4206	0.004

Smaller values of Observe-delta indicate smaller within-group differences, and larger values of Expect-delta indicate larger between-group differences. A > 0, Between-group differences were greater than within-group differences; A < 0, Differences within groups were greater than differences between groups. Significance < 0.05, the difference is significant.

### 3.3 *Pseudomonas* dominance in phyllosphere bacteria, and symptom-linked *Xanthomonas* enrichment

**Figure 3:**
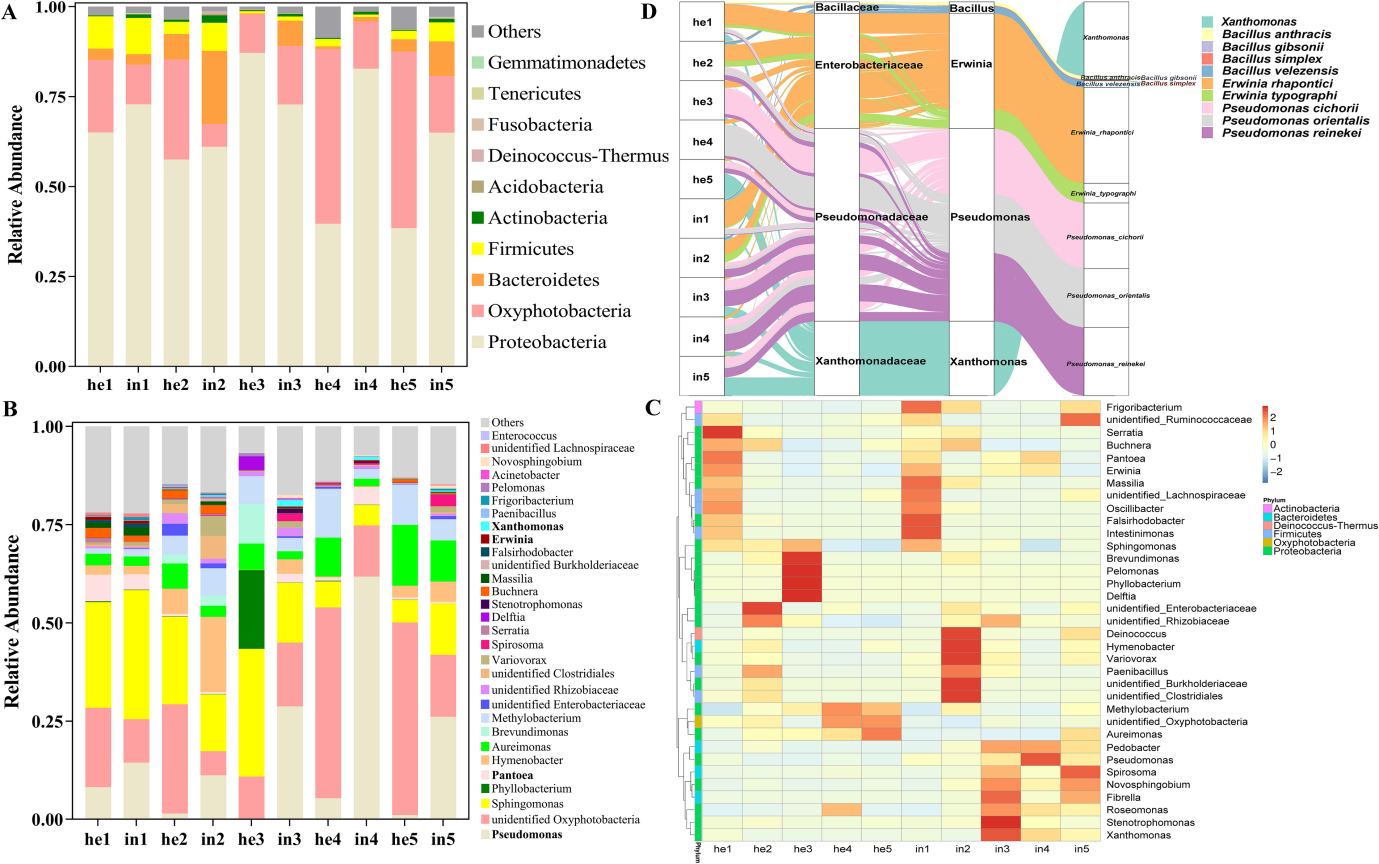
Abundance and composition of phyllosphere microbe. Relative abundance by bacterial group analysis of **(A)** the top 10 at the phylum level, **(B)** the top 35 at the genus level. Heatmap clustering of bacteria **(C)** at the genus level, cluster analysis was based on the Bray–Curtis method and only the top 35 abundant genera were included. Columns represent different sample groups, and rows represent different genera. On the left is the hierarchical clustering at the phylum level, light-pink, cyan-blue, light-coral, light-blue, light-yellow, and light-green represent Actinobacteria, Bacteroidetes, Deinococcus-Thermus, Firmicutes, Oxyphotobacteria, and Proteobacteria, respectively. Color represents relative abundance (by Z-score) from low (blue, −2) to high (red, 2). Sankey Diagram **(D)**, the classification of microorganisms at different levels; the three columns represent the three levels of family-genus-species.

We analyzed the relative abundance of bacterial species to assess community composition across conditions. At the phylum level, the dominant ones for the symptomatic groups (in1–in5) and asymptomatic groups (he1–he5) mainly included Proteobacteria, Oxyphotobacteria, and Bacteroidetes (Figure 3A). At the genus level, the relative abundance of *Pseudomonas* spp. and *Xanthomonas* spp. was significantly higher in in3, in4, in5 than in he3, he4, he5 (Figures 3B,C). The relative abundance of *Pseudomonas* spp. increased 237.79-fold, and that of *Xanthomonas* spp. increased 289.93-fold in the third sample (Figures 3B,C; Supplementary Table SIII).

Sankey diagram was used to represent the pathogenic bacteria and biocontrol potential bacteria of walnut leaf spotting diseases. The relative abundance of *Xanthomonas* spp. was higher in the symptomatic groups (in) than in the asymptomatic groups (he) throughout the sampling cycle, reaching a maximum in the third sampling time and gradually decreasing with the disease progression. *Pseudomonas* spp. occupied the largest proportion of all samples, and the relative abundance of *Erwinia* spp. was slightly higher than that of *Xanthomonas* spp. in both symptomatic and asymptomatic groups; the relative abundance of *Xanthomonas* spp. in symptomatic groups was higher than that in asymptomatic groups during the same sampling time (Figure 3D; Supplementary Table SIII).

### 3.4 *Pseudomonas* as keystone taxa bridging microbiome reassembly and walnut pathogen network

**Figure 4:**
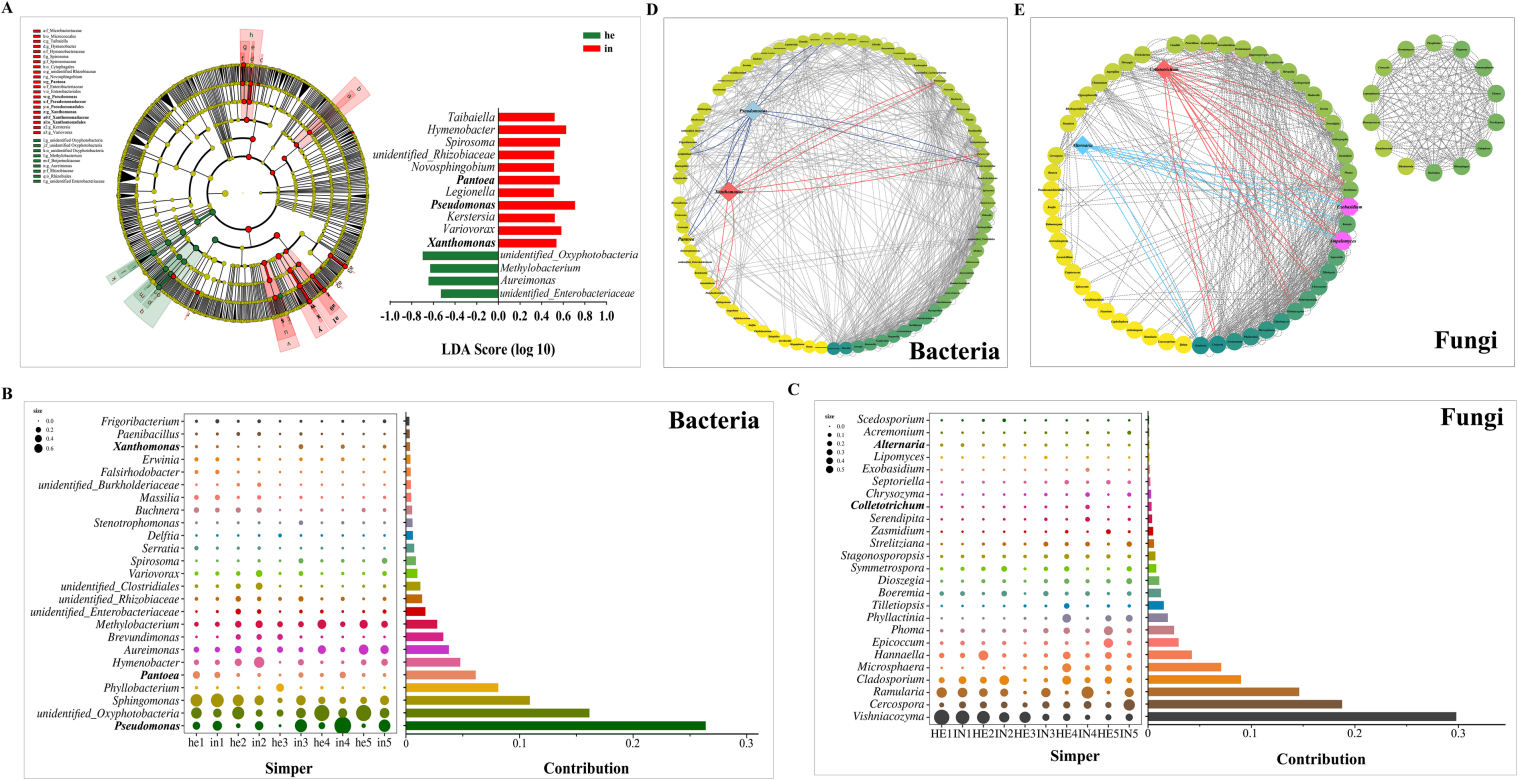
Differences in phyllosphere microbial community structure of walnut leaf in different groups. **(A)** Cladograms of linear discriminant analysis coupled with the effect size analysis with logarithmic linear discriminant analysis >3.25 (*p* < 0.05) reveals the differences in abundant taxa of phyllosphere bacterial communities between symptomatic and asymptomatic group at phylum to genus levels. **(B,C)** Contribution of phyllosphere bacteria and fungi at the genus levels to intergroup differences between symptomatic and asymptomatic walnut leaf, similarity percentage was based on Bray–Curtis distances and only the top 25 genera were included. Bubble size represents the relative abundance of the species, Contribution is the value of the species’ contribution to the variability of the symptomatic and asymptomatic group. **(D,E)** Genus-level species correlation network diagram, the top 100 at the genus level were calculated based on Spearman correlation coefficients, with correlation coefficients greater than 0.6 in absolute value and *p* < 0.05 as valid connections. Node color represents connectivity at the genus level from low (lighter) to high (darker).

Phyllosphere bacterial microbial taxa from phylum to genus were identified between symptomatic and asymptomatic walnut leaf groups using linear discriminant analysis and the effect size analysis. Throughout the sampling cycle, the microbial communities of the symptomatic groups were significantly (*p* < 0.05) altered compared to the asymptomatic groups (Figure 4A; Table 2); it is characterized by the high relative abundance of 11 genera, including *Xanthomonas* spp., *Pseudomonas* spp., and *Pantoea* spp., which play an important role as biomarkers in the symptomatic phyllosphere microbiome. While unidentified *Oxyphotobacteria*, *Methylobaterium*, *Aureimonas*, and unidentified *Enterobacteriaceae* as biomarkers in the asymptomatic group. Among them, the symptomatic groups were most affected by *Pseudomonas* spp., and *Oxyphotobacteria* spp. had the maximum LDA score among the asymptomatic groups.

Similarity percentage (SIMPER) was used to identify the major species driving phyllosphere microbial community patterns. The analysis of phyllosphere bacteria between asymptomatic and symptomatic groups (he vs. in) showed that *Pseudomonas* spp. ranked first and accounted for 26.38% of the total Bray-Curtis difference between symptomatic and asymptomatic groups, *Pantoea* spp. accounted for 6.14%, *Xanthomonas* spp. accounted for 0.35% (Figure 4B). In comparing fungal asymptomatic and symptomatic groups (HE vs. IN), *Vishniacozyma* spp. contributed the highest value, while the *Cladosporium* spp., *Colletotrichum* spp., and *Alternaria* spp. contributed 8.98, 0.33, and 0.11%, respectively, of the total difference (Figure 4C).

A species correlation network diagram of the asymptomatic and symptomatic leaf group’s core genera was constructed. The asymptomatic and symptomatic phyllosphere microbiomes were identified to be related to each other, forming co-occurrence networks (correlation coefficients greater than 0.6 in absolute value and *p* < 0.05; Figures 4D,E; Supplementary Tables SV, SVI). It showed that *Xanthomonas* spp. with *Pseudomonas* spp., *Pseudarthrobacter* spp., *Pelomonas* spp. and *Paracoccus* spp., *Pseudomonas* spp. with *Pantoea* spp. had valid connections (*p* < 0.05). Besides, both the *Colletotrichum* spp. and *Alternaria* spp. had valid connections with *Exobasidium* spp., *Ampelomyces* spp.

### 3.5 Symptomatic phyllosphere microbial functional shift: pathogen-linked fungi and metabolic bacteria enrichment

**Figure 5:**
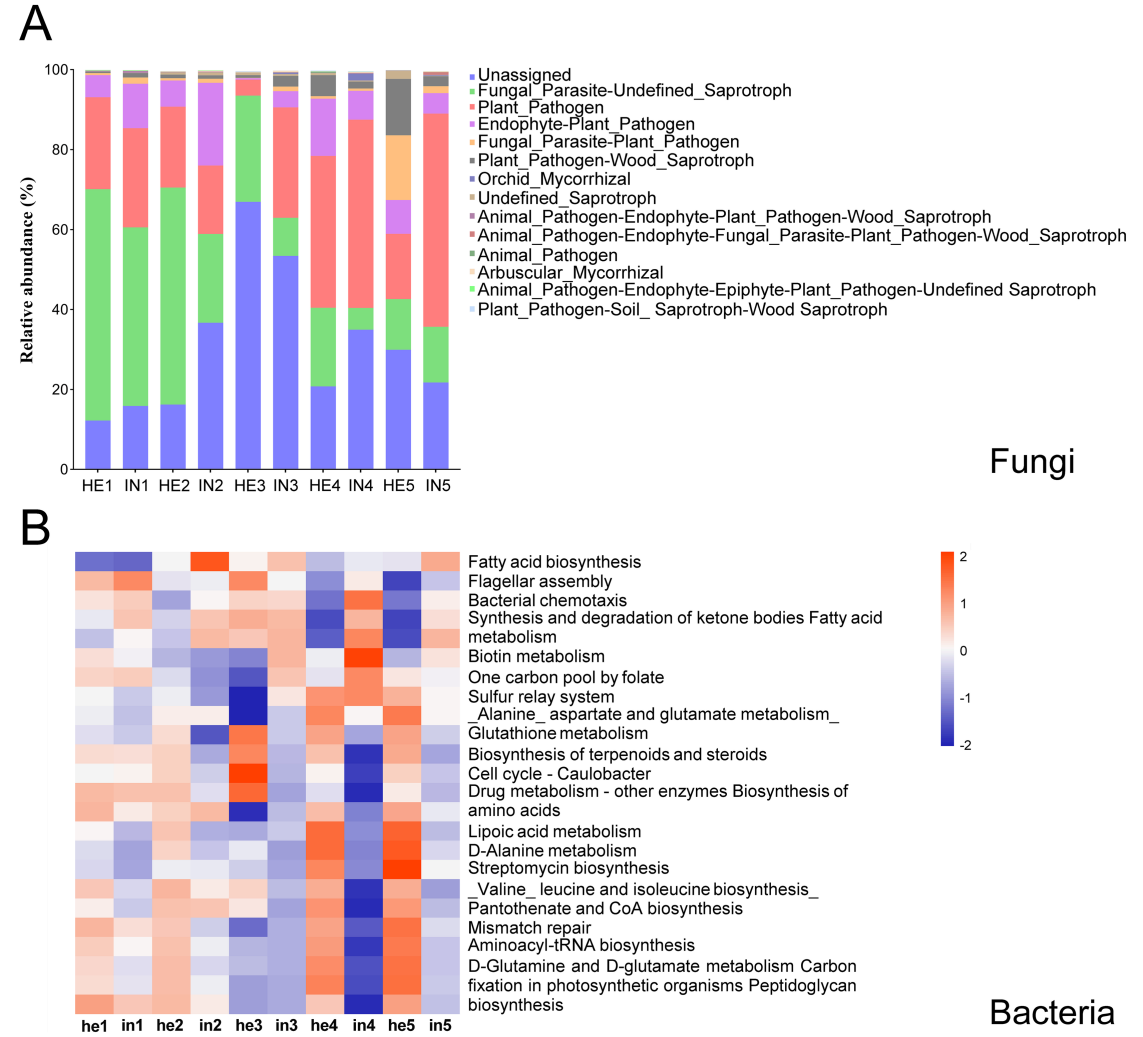
Functional predictions of phyllospheric fungal and bacterial communities. **(A)** Variations in compositions, predicted by FUNGuild, fungal functional groups. **(B)** Heatmap of the top 25 abundant bacterial functions in the KEGG pathway, predicted by PICRUSt2, red represents high relative abundance of functional pathways, while blue represents low relative abundance. Columns represent different sample groups, and rows represent different functional pathways.

The FUNGuild analysis showed that the relative abundance of fungal taxa associated with Plant_Pathogen functions in the symptomatic walnut phyllosphere was increased by up to 30.33% higher than the asymptomatic phyllosphere during the sampling period. In contrast, asymptomatic fungi exhibited a higher relative abundance of taxa linked to Fungal Parasite-Undefined Saprotroph functions from the first to fourth sampling time (Figure 5A; Supplementary Figure S2). Additionally, fungal taxa related to Fungal_Parasite functions in the symptomatic group showed a 12.17 to 31.65% lower relative abundance compared to the asymptomatic group (Figure 5A; Supplementary Figure S2). On the other hand, the PICRUSt2 analysis indicated that infection increased the relative abundance of phyllosphere bacterial taxa associated with the KEGG pathway functions of Fatty acid biosynthesis and Biotin metabolism. Conversely, asymptomatic groups accumulated more bacterial taxa enriched in Cell cycle -Caulobacter and Streptomycin biosynthesis functions than symptomatic groups (Figure 5B; Supplementary Figure S2).

### 3.6 Dew point temperature and liquid precipitation depth dimension, respectively, dominated phyllosphere bacteria and fungi

**Figure 6:**
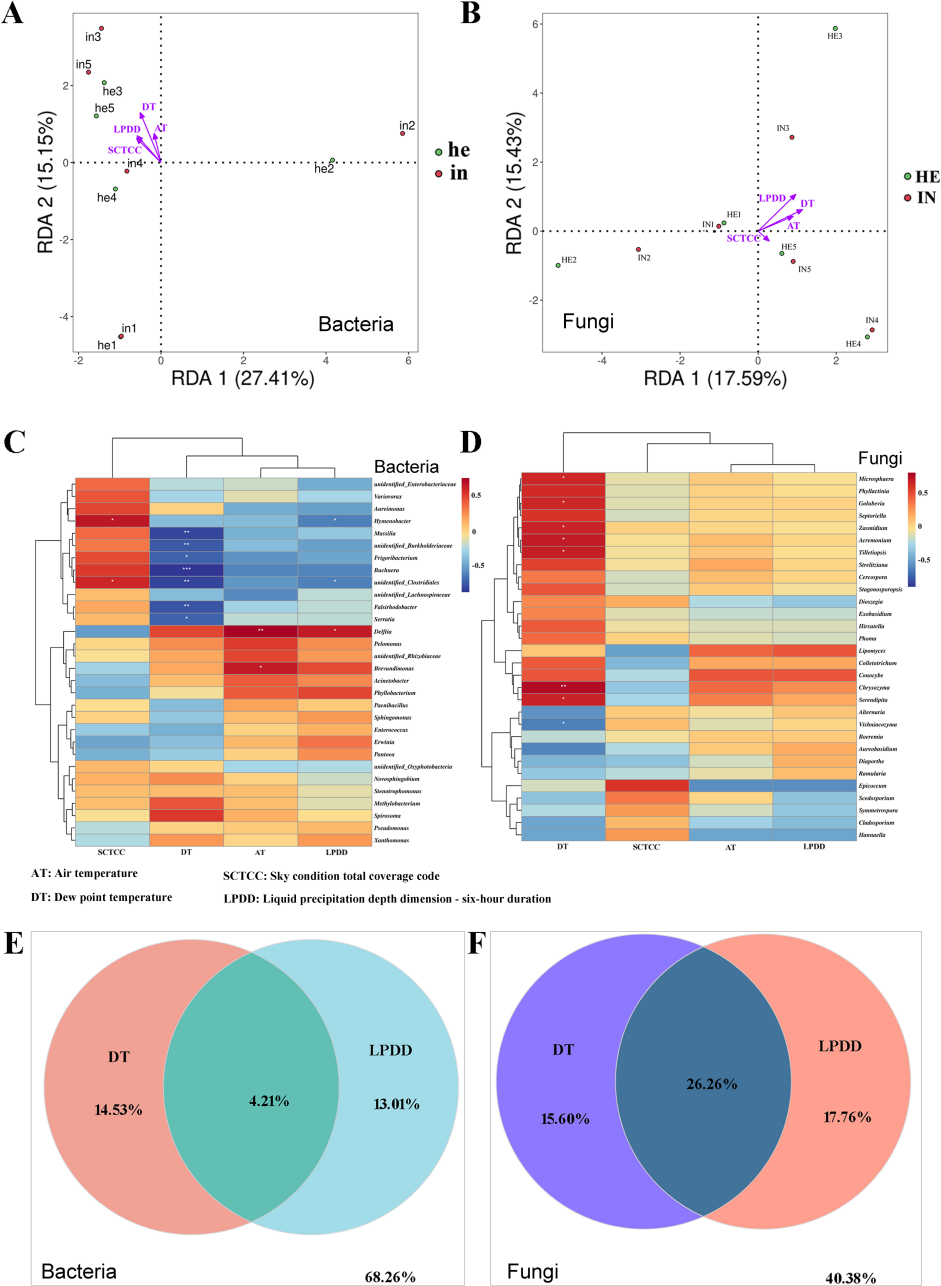
Redundancy analysis (RDA), Spearman correlation heatmaps, and Variance partitioning analysis (VPA) for revealing the relationships between the relative abundance of key microbes at the genus level and environmental factors (Air temperature, AT; dew point temperature, DT; sky condition total coverage code, SCTCC; liquid precipitation depth dimension - six-hour duration, LPDD) of the symptomatic and asymptomatic group. **(A,B)** RDA of correlation between phyllosphere bacteria and fungi relative abundance and environmental factors; **(C,D)** Heatmaps of relationships between phyllosphere bacterial and fungal taxas relative abundance and environmental factors, columns represent different environmental factors, and rows represent different genera, and color from blue to red represents Spearman’s rank correlation coefficient (rho) from −1 to 1, *0.01 < *p* ≤ 0.05; **0.001 < *p* ≤ 0.01; ***p ≤ 0.001; **(E,F)** VPA of contributions of dew point temperature and iquid precipitation depth dimension to changes in phyllosphere bacterial and fungal communities in walnut.

Redundancy analysis (RDA) was used to investigate the effects of abiotic factors, including Air temperature (AT), dew point temperature (DT), sky condition total coverage code (SCTCC), and liquid precipitation depth dimension (LPDD) on the phyllosphere microbial taxa of symptomatic and asymptomatic walnut leaves. According to the sampling time, the data of environmental factors in that time range were recorded (Supplementary Table SIII). RDA results of bacterial and fungal communities with environmental factors revealed a positive correlation between each factor. The correlation between dew point temperature and bacterial community and species distribution was the highest, followed by liquid precipitation depth dimension and the lowest correlation with air temperature (Figure 6A). The correlation between liquid precipitation depth dimension and fungal community and species distribution was the highest, dew point temperature the second highest, and sky condition total coverage code the lowest (Figure 6B).

Further, the mantel-test was used to analyze the correlation between these factors and microbial communities (Table 3). Significant correlations (*p* < 0.05) between all four environmental factors and bacterial/fungal community species distribution were found. While liquid precipitation depth dimension (*r* = 0.2189, *p* = 0.001) and dew point temperature (*r* = 0.2058, *p* = 0.001) were highly correlated with bacterial community species abundance, fungal community species abundance was highly correlated with sky condition total coverage code (*r* = 0.3753, *p* = 0.001) and liquid precipitation depth dimension (*r* = 0.2553, *p* = 0.001).

**Table 3:** Mantel-test calculations of walnut phyllosphere bacterial and fungal communities in relation to environmental factors.

Mantel r, P	Air temperature (AT)	Dew point temperature (DT)	Sky condition total coverage code (SCTCC)	Liquid precipitation depth dimension—6 h duration (LPDD)
Bacterial 16S	0.1541, 0.003	0.2058, 0.001	0.1895, 0.001	0.2189, 0.001
Fungal ITS	0.2084, 0.001	0.18, 0.001	0.3753, 0.001	0.2553, 0.001

The larger the *r*-value, the greater the correlation between the environmental factor and the species abundance information of the group; *p* < 0.05, then there is a significant correlation between the environmental factor and the distribution of microbial community species.

The heatmap clustering examined the difference in the phyllosphere’s top 30 abundant bacterial and fungal genera. The phyllosphere bacteria genera *Massilia*, *unidentifed_Burkholderiaceae*, *Frigoribacterium*, *Buchnera*, *unidentifed_Clostridiales*, *Falshirodobacteria*, and *Serratia*, were all negatively correlated with the dew point temperature. The genus *unidentifed_Clostridiales* is also negatively correlated with liquid precipitation depth dimension but positively correlated with sky condition total coverage code. The genus *Delftia* was positively correlated with air temperature and liquid precipitation depth dimension, and the genus *Hymenobacter* was negatively correlated with liquid precipitation depth dimension but positively correlated with sky condition total coverage code (Figure 6C). As for the fungal taxa, *Microsphaera*, *Golubevia*, *Zasmidium*, *Acremonium*, *Tilletiopsis*, *Chrysozyma*, and *Serendipita* were all positively correlated with dew point temperature, and *Vishniacozyma* was negatively correlated (Figure 6D).

Dew point temperature and liquid precipitation depth dimension were selected for variance partitioning analysis to understand the contribution of the two environmental factors individually and in interaction with the microbial community changes, respectively. The results showed that the interaction of dew point temperature and liquid precipitation depth dimension contributed more to changes in both bacterial and fungal communities than the two factors alone, and the contribution of the interaction was more pronounced in the fungal community (Figures 6E,F).

### 3.7 Pathogen-driven photosynthetic suppression shows fungal Pn linkage and bacterial Pn & Gs ties

**Figure 7:**
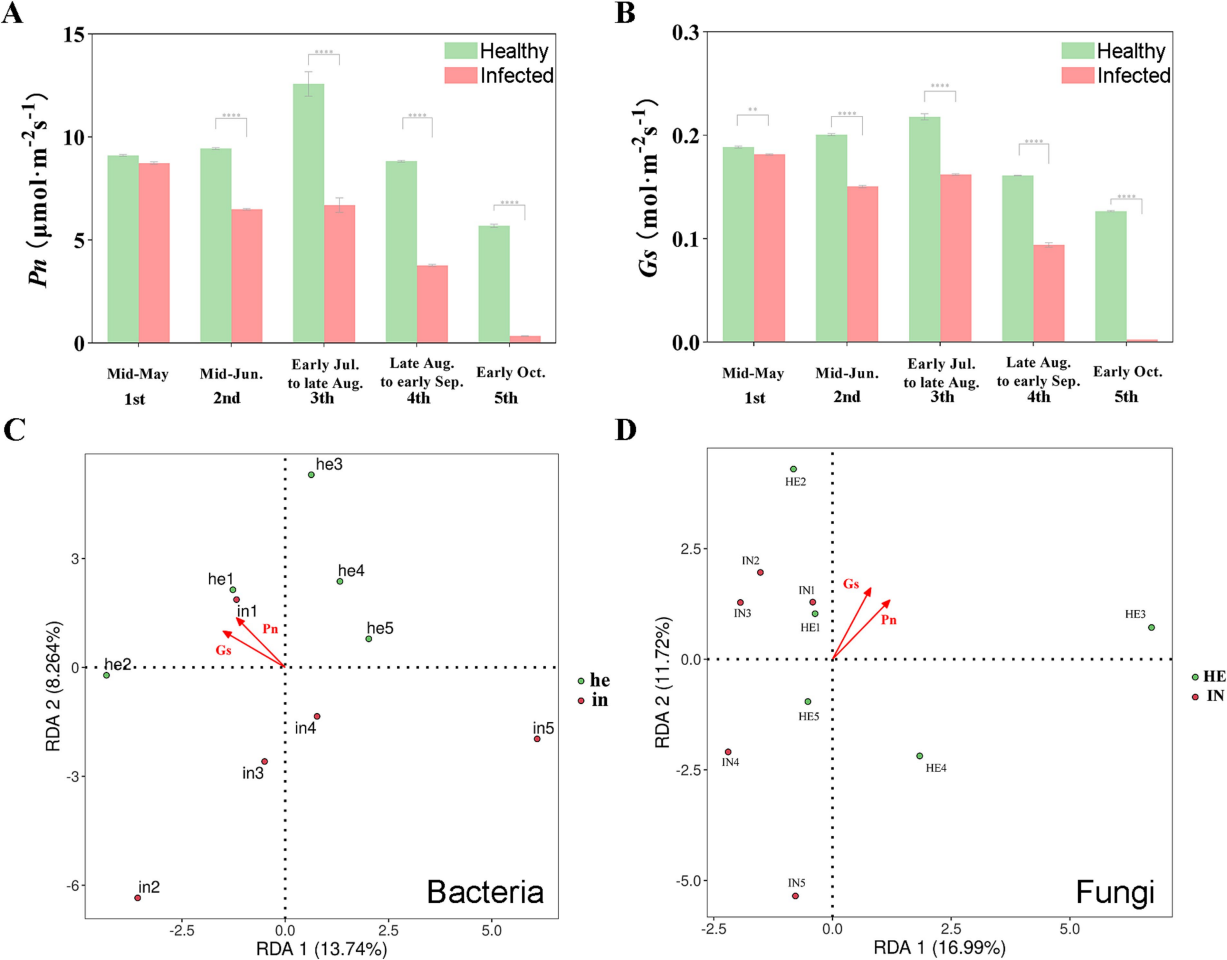
The relationships between the relative abundance at the genus level and host plant performances of the symptomatic and asymptomatic group. **(A)** Net photosynthetic rate (Pn) of symptomatic and asymptomatic walnut leaves during the sampling times; **(B)** Stomatal conductance (Gs) of symptomatic and asymptomatic walnut leaves during the sampling times; **(C,D)** Redundancy analysis (RDA) of correlation between phyllosphere bacteria and fungi relative abundance and walnut leaves performances (Pn and Gs). *0.01 < *p* ≤ 0.05; **0.001 < *p* ≤ 0.01; ***p ≤ 0.001.

Symptomatic leaves showed significant decreases in net photosynthetic rate and stomatal conductance compared to asymptomatic leaves, except at the first sampling time (Figures 7A,B). The effect of leaf photosynthetic characteristics on the phyllosphere microbial community was investigated by redundancy analysis (RDA). Significant correlation showed between bacterial community and net photosynthetic rate (R^2^ = 0. 0.8768, *p* = 0.002) and stomatal conductance (R^2^ = 0.9063, *p* = 0.002; Figure 7C). As for fungal community, it was significantly correlated with net photosynthetic rate (R^2^ = 0.8895, *p* = 0.002) and slightly weaker correlation with stomatal conductance (R^2^ = 0.8737, *p* = 0.005; Figure 7D).

Redundancy analysis (RDA) revealed the integrated environment-plant performance model with single-factor models demonstrated higher explanatory power for microbial community variation. The cumulative explanatory proportions of RDA1 and RDA2 reached 36.9 and 43.58%, respectively, in the integrated model of fungal and bacterial communities. In contrast, single-factor models showed lower cumulative proportions: environmental parameters alone accounted for 33.02% (fungi) and 42.56% (bacteria) of variation, while plant performance traits alone explained 28.71% (fungi) and 22.004% (bacteria), respectively. These results indicate that the synergistic consideration of environmental parameters (AT, DT, SCTCC, LPDD) and plant physiological traits (Pn, Gs) exerts stronger influences on phyllosphere microbial communities than individual factor analyses (Supplementary Figures S4A,B; Figures 6A,B, 7C,D). Notably, net photosynthetic rate (Pn) emerged as the most significant factor for fungal community composition among the explanatory variables. In contrast, dew point temperature (DT) showed predominant influence on bacterial community assembly (Supplementary Figures S4A,B).

## 4 Discussion

In this study, asymptomatic and symptomatic walnut leaves from different phenological periods were collected to explore the differences in the phyllosphere microbial community by amplifying the V4 region of 16S rRNA and the ITS region of rDNA. Previous research has consistently demonstrated that phyllosphere microbes’ populations change during plant-pathogen interactions and that the phyllosphere community undergoes changes when pathogens infect leaves. It is generally agreed that communities with stable configurations and high species richness exhibit greater resistance to pathogen invasion (Wei et al., 2015; Liu et al., 2024). Our results showed that the phyllosphere microbial bacterial community richness in asymptomatic walnut leaves was lowest in the third sampling (early Jul. to late Aug.). In contrast, fungal community richness and diversity were lowest in the third sampling data. Further to the prediction of the function of the phyllosphere microbial community, there were far more fungal taxa associated with Fungal_Pathogen in symptomatic leaves (IN3) than in asymptomatic leaves (HE3; Figure 5A). This evidence supports a greater likelihood of leaf spotting disease at the fruit swelling stage. The peak of community distance between symptomatic and asymptomatic leaves also appeared in the third sampling (Supplementary Figure S3), and combined with the area of leaf spotting disease (Supplementary Table SI), changes in the abundance and diversity of the phyllosphere microbial community coincided with the peak severity of disease occurrence. As phenological stages progressed, fungal communities transitioned from a dispersed state to relative clustering in the late sampling times, whereas bacterial communities maintained a relative dispersed distribution (Supplementary Figure S3). This observation aligns with evidence suggesting bacterial communities exhibit higher sensitivity to phenological shifts, potentially due to their faster metabolic responses to resource fluctuations (Wujisiguleng et al., 2023). However, the results of our previous analyses corroborate that the phyllosphere fungal community of symptomatic walnuts exhibited higher richness (as measured by the Chao1 index) and greater diversity (as indicated by the Shannon index) compared to asymptomatic leaves at the same time point (Wang et al., 2022). Similar results were obtained in this work for higher bacterial community richness and diversity under leaf infection (Figure 2); this is consistent with the trend of a significant increase in the phyllosphere bacterial and fungal Shannon and Chao1 index in infected *Pinus koraiensis* (Deng et al., 2021). This may be related to pathogen invasion disrupts leaf surface integrity through mechanical wounding and enzymatic degradation, creating entry points for microbial colonization, and this physical damage facilitates intracellular nutrient leakage while suppressing plant immune responses (Xiong et al., 2021; Zhang et al., 2023).

We found a significant effect of disease on the diversity of phyllosphere microbial bacterial communities (Table 2). It is noteworthy that Firmicutes had lower abundance and Proteobacteria had higher abundance in the third and fourth samples with severe walnut leaf disease (Figure 3A). Numerous studies have demonstrated that changes in the abundance of Firmicutes and Proteobacteria are closely related to the ecological dysbiosis of the phyllosphere microbiota (Gong and Xin, 2021), showing a shift from a Firmicutes phylum rich community to a Proteobacteria phylum rich community (Lee et al., 2021; Runge et al., 2023; Sun et al., 2023). Meanwhile, we found that the relative abundance of *Bacillus* spp. was higher in the phyllosphere microbial community of asymptomatic leaves than that of symptomatic leaves (Figure 3D). Further, *Bacillus gibsonii* and *Bacillus velezensis* are reported antagonistic bacteria against the pathogen of maize and walnut in our species level results (Batool et al., 2019; Wang and Zhu, 2023). *Bacillus* spp. have been shown to exhibit antagonistic activity against *Colletotrchum* spp., *Xanthomonas* spp. and also play a positive role in walnut leaf homeostasis (Srikhong et al., 2018; Marin et al., 2019). Interestingly, we noted that *Pseudomonas* spp. was also present in asymptomatic leaves (Figure 3D); *Pseudomonas orientalis* is an antagonist of the apple flower against phytopathogens but *Pseudomonas cichorii* caused tobacco, rromaine lettuce, escarole, tomato, lettuce and endive leaf spot diease (Zengerer et al., 2018; Arshad et al., 2021; Lan et al., 2022; Patel et al., 2021; Osdaghi, 2022).

Phyllosphere microbial communities are influenced by abiotic and host factors that act directly on hub taxa (Agler et al., 2016). We identified the primary bacterium driving the microbial community pattern of symptomatic and asymptomatic walnut leaves as the genus *Pseudomonas* by similarity percentage, which is also included in the plant-associated microbial keystone taxa summarized by Banerjee et al. (2018). Other pathogenic bacteria (*Xanthomonas* spp., *Pantoea* spp.) and pathogenic fungi (*Cladosporium* spp., *Colletotrichum* spp., *Alternaria* spp.) of walnut leaf spotting diseases were among the top 25 genera in their respective contribution values (Figures 4B,C). The pathogen *Exobasidium* causes leaf diseases of *Camellia Oleifera* and rabbiteye blueberry (Ingram et al., 2019; Zhang et al., 2022), but its pathogenicity in walnut has not been assessed. This study revealed the existence of valid connections with the identified leaf spotting disease pathogenic fungus, *Colletotrichum* and *Alternaria* (Figure 4E). Studies on other plants show that *Pseudomonas* spp. can self-aggregate and co-aggregate with *Xanthomonas* spp. (Afkhamifar et al., 2023). Our results also demonstrate an interaction between the two (Figure 4D), which can correlate with walnut leaf diseases showing a multi-pathogen complex infestation and multiple diseases interacting under natural conditions. In previous studies, the main causal agent of the walnut black spot was *Xanthomonas* (Lamichhane, 2014), but our results suggest that when targeting walnut leaf spotting diseases for prevention and control, it could be centered around *Pseudomonas* (Figures 4A,B,D), perhaps with better results, but further research is needed to find out exactly what effects are produced between the several pathogens. On the other hand, microbial community profiling identified *Oxyphotobacteria*, *Methylobaterium*, *Aureimonas*, and unidentified *Enterobacteriaceae* as biomarkers in asymptomatic walnut leaves phyllosphere microbiome, particularly during the third to fifth sampling times (Figures 4A,B). The sustained dominance of *Methylobacterium* (a known phyllosphere mutualist) and Oxyphotobacteria suggests their synergistic roles in host protection. These taxa may enhance plant firness through synergistic roles in host protection and promote plant growth (Zhu et al., 2023; Zhang et al., 2024), potentially explaining their association with the health of asymptomatic plants.

Since seasonal variation is centered on changes in a number of environmental factors, including temperature, humidity, and light, we investigate the relationship between these abiotic factors and the phyllosphere microbial community. The results showed that air temperature (AT), dew point temperature (DT), sky condition total coverage code (SCTCC) and liquid precipitation depth dimension (LPDD) were all significantly correlated with the phyllosphere microbial community, with DT and LPDD being the most highly correlated factors for bacterial and fungal communities, respectively (Figures 6A,B). As for fungi, the genera *Microsphaera*, *Golubevia*, *Zasmidium*, and *Tilletiopsis*, which showed significant positive correlation with dew point temperature (Figure 6D), include plant pathogens and may cause powdery mildew, oil spot, and others (Abasova et al., 2017; Richter et al., 2019; Afkhamifar et al., 2023; Aguilera-Cogley et al., 2023; Guarnaccia et al., 2024). *Xanthomonas* spp. and *Pseudomonas* spp. were all positively correlated with LPDD (Figure 6C), which is consistent with the effect of rainfall on the relative abundance of both genera of bacteria in the epiphytic bacterial communities of cucumber and tomato (Allard et al., 2020). The correlation with precipitation may be related to the spread of phyllosphere bacteria through rain splash or windblown rain (Bahadur and Srivastava, 2022), and the increase in water utilization by *Pseudomonas* spp. through the release of surfactants (Hernandez and Lindow, 2019). Throughout the sampling period, the third sampling time was the highest temperature, and the fourth was the next highest. The relative abundance of *Xanthomonas*, *Pseudomonas*, and *Erwinia* was elevated at this time (Figures 3B,D), in agreement with Aydogan et al. (2020) on the effect of elevated temperature on the relative abundance of potential phyllosphere pathogens as well.

In addition to abiotic factors, the state of the plant itself influences the phyllosphere microbial community. We investigated the relationship between net photosynthetic rate, stomatal conductance, and the phyllosphere microbial community. Walnut leaf infections significantly reduced net photosynthetic rate and stomatal conductance, which was consistent with the trend of reduced stomatal conductance after *Quercus robur* (Hajji et al., 2009). *Xanthomonas* and *Pseudomonas* tend to enter plant stems, leaves, or floral tissues through wounds or natural openings (e.g., stomata or hydathodes; Gudesblat et al., 2009). Li et al. (2021) showed that chlorophyll and plant biomass influence the diversity of the phyllosphere microbial community, and both factors are positively correlated with leaf photosynthesis. Through redundancy analysis (RDA), we found that bacterial and fungal communities significantly correlated with photosynthetic properties (Figures 7C,D), demonstrating that phyllosphere microbes and leaves influence and regulate each other. Redundancy analysis (RDA) of the environment and plant performance model identified net photosynthetic rate (Pn) and dew point temperature (DPT) as key drivers shaping phyllosphere fungal and bacterial community composition, respectively (Supplementary Figure S4). Mechanistically, the net photosynthetic rate fluctuations may regulate fungal communities by changing the quantity and quality of carbon sources plants provide to fungi (Chen et al., 2024). In contrast, dew point temperature likely influences bacterial communities by affecting the water vapor content and condensation in the phyllosphere (Gao et al., 2016).

## 5 Conclusion

This study revealed dynamic shifts in the phyllosphere microbial communities of walnut leaves during phenological progression, driven by pathogen infection, environmental factors, and host physiological traits. These results demonstrated that symptomatic leaves exhibited significantly higher bacterial richness and diversity than asymptomatic leaves. The observed differences were correlated with disease severity during phenology progression. Notably, symptomatic leaves were characterized by *Pseudomonas* spp. (a keystone species) forming ecological associations with other pathogens (*Xanthomonas* and *Pantoea*) associated with walnut leaf spotting disease. In contrast, asymptomatic leaves predominantly harbored beneficial taxa including *Methylobacterium* and *Oxyphotobacteria*. Environmental factors, particularly dew point temperature (DT) and liquid precipitation depth (LPDD), significantly influenced phyllosphere community, with their interaction exerting stronger effects than individual factors. Net photosynthetic rate (Pn) and dew-point temperature (DT) are the most prominent factors exerting influence on fungal and bacterial communities under integrated environment-plant performance model, highlighting bidirectional plant-microbe interactions. These findings reveal the dynamic complexity of phyllosphere microbial ecology in disease contexts, shaped by microbial interactions and environmental-physiological factor synergies. While this study provides insights into microbial shifts during walnut leaf spot disease, limitations are unresolved pathogenicity mechanisms that warrant further investigation. Future research should prioritize targeted biocontrol strategies leveraging antagonistic taxa (e.g., *Bacillus* spp.) and explore multi-omics approaches to decipher functional pathways underlying microbial-plant-environment interactions.

## Data Availability

The datasets presented in this study can be found in online repositories. The names of the repository/repositories and accession number(s) can be found in the article/Supplementary material.

## Footnotes

^1^ http://ccb.jhu.edu/software/FLASH/

^2^ http://drive5.com/uparse/

^3^ http://www.arb-silva.de

^4^ https://www.ncdc.noaa.gov

^5^ www.Bioincloud.tech

^6^ https://doi.org/10.33073/pjm-2022-038
