# Supplementary material for: Temporal dynamics of walnut phyllosphere microbiota under synergistic pathogen exposure and environmental perturbation
Source: Front Microbiol. 2025 Apr 1;16:1551476. doi: 10.3389/fmicb.2025.1551476 (PMC11996876; doi:10.3389/fmicb.2025.1551476)
Supplement: Supplementary file 1 [file Data_Sheet_1.pdf]

Supplementary information

The following Supporting data is available for this article:

Fig. S1 Walnut leaves

(A), Asymptomatic leaves from the third sampling; (B), Symptomatic leaves from the third sampling.

Fig. S2 Relative abundance of fungal functional compositions, predicted by FUNGuild

Blue solid lines indicate asymptomatic walnut (HE) phyllosphere fungal taxa related to the function including Plant_Pathogen; blue dashed lines indicate symptomatic walnut (IN) phyllosphere fungal taxa related to the function including Plant_Pathogen. Orange solid lines indicate asymptomatic walnut (HE) phyllosphere fungal taxa related to the function including Fungal_Parasite; Orange solid lines indicate symptomatic walnut (IN) phyllosphere fungal taxa related to the function including Fungal_Parasite.

Fig. S3 Non-metric Multidimensional Scaling (NMDS) plot at the genus level

(A), NMDS of phyllosphere bacteria; (B), NMDS of phyllosphere fungi.

Fig. S4 Redundancy analysis (RDA) of correlation between phyllosphere microbe relative abundance and environment-plant performance model

(A), RDA of phyllosphere bacteria; (B), RDA of phyllosphere fungi.

Table SI. Sample name and sampling time

Table SII. Statistical table of 16S rDNA Amplicon Sequencing Alpha diversity index (10 groups)

Table SIII. Table SIII Relative abundance by bacterial group anallysis of the top35 at the genus level

Table SIV. Environmental conditions during the period of sampling

Table SV. Spearman correlation table of phyllosphere bacterial genera to one another

Tabel SVI. Spearman correlation table of phyllosphere fungal genera to one another

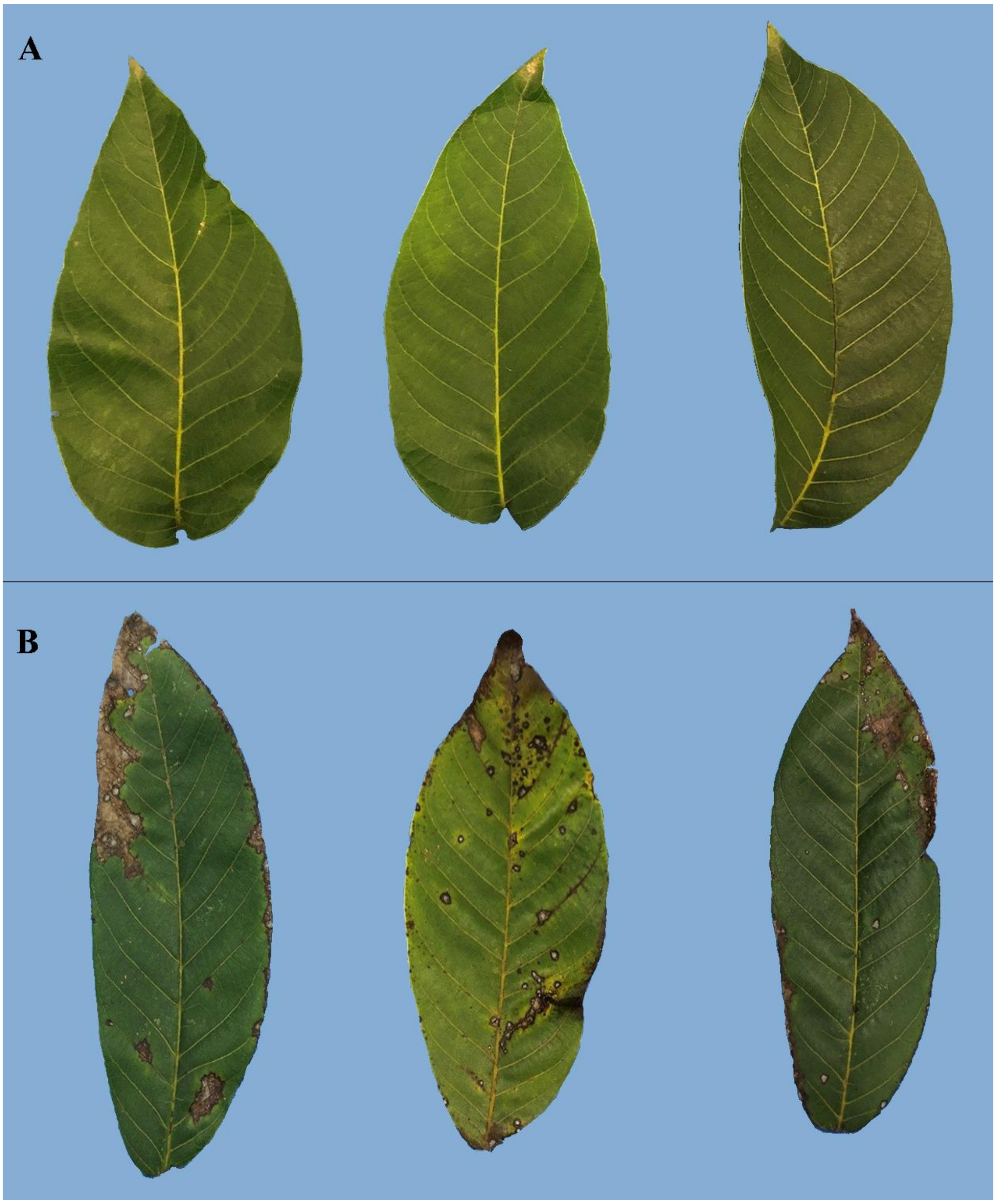

Fig. S1 Walnut leaves

(A), Asymptomatic leaves from the third sampling; (B), Symptomatic leaves from the third sampling.

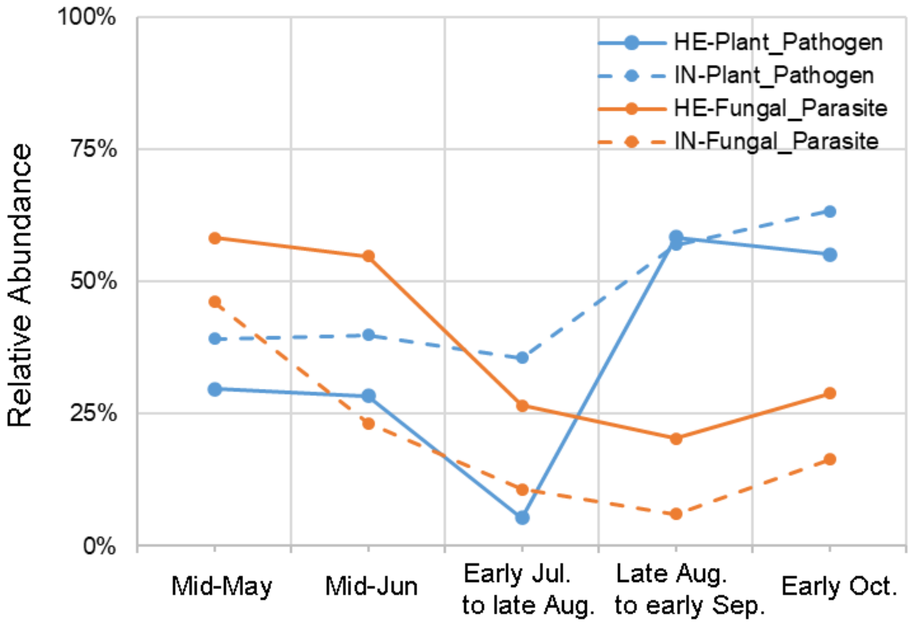

Fig. S2 Relative abundance of fungal functional compositions, predicted by FUNGuild

Blue solid lines indicate asymptomatic walnut (HE) phyllosphere fungal taxa related to the function including Plant_Pathogen; blue dashed lines indicate symptomatic walnut (IN) phyllosphere fungal taxa related to the function including Plant_Pathogen. Orange solid lines indicate asymptomatic walnut (HE) phyllosphere fungal taxa related to the function including Fungal_Parasite; Orange solid lines indicate symptomatic walnut (IN) phyllosphere fungal taxa related to the function including Fungal_Parasite.

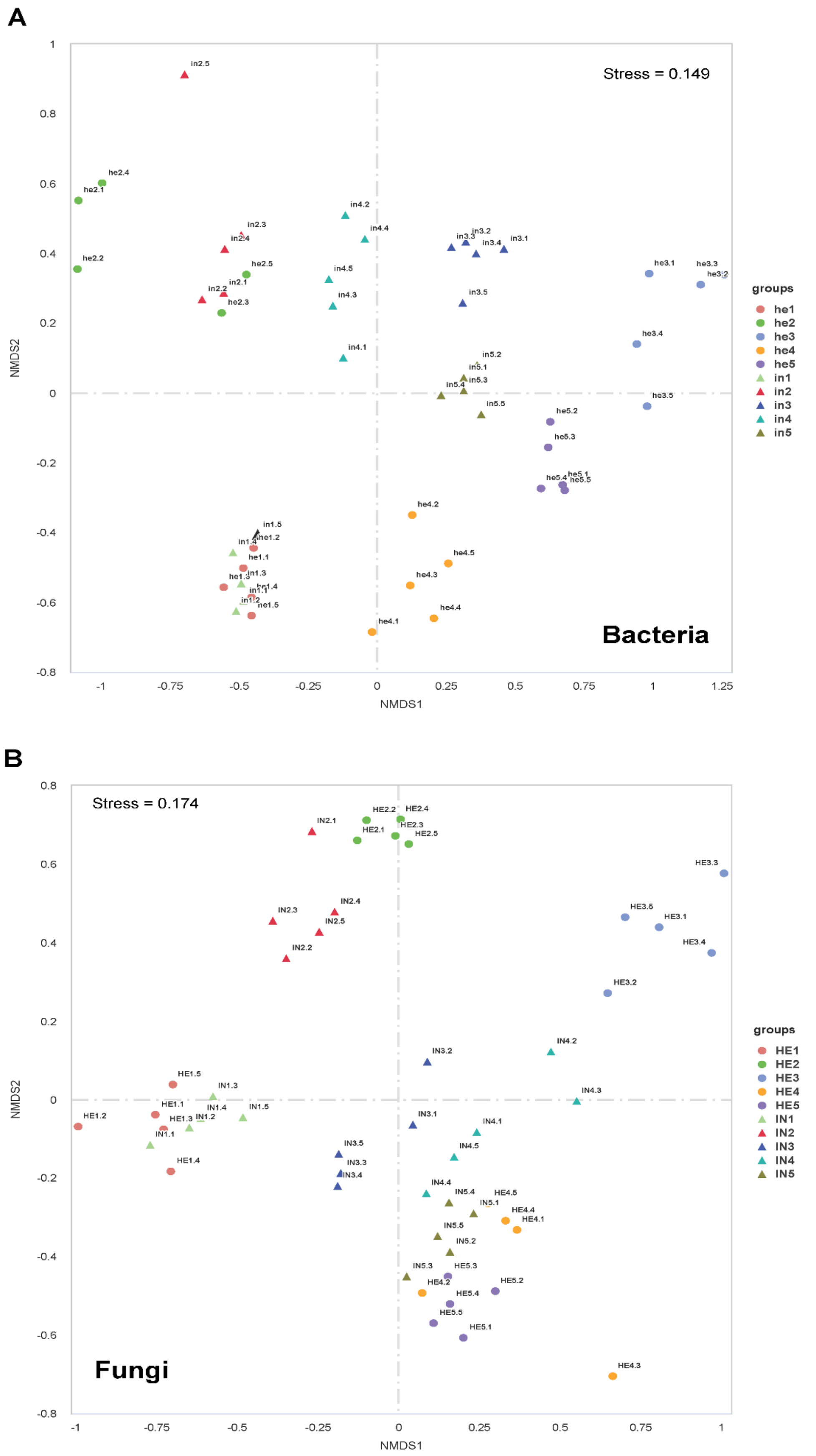

Fig. S3 Non-metric Multidimensional Scaling (NMDS) plot at the genus level

(A), NMDS of phyllosphere bacteria; (B), NMDS of phyllosphere fungi.

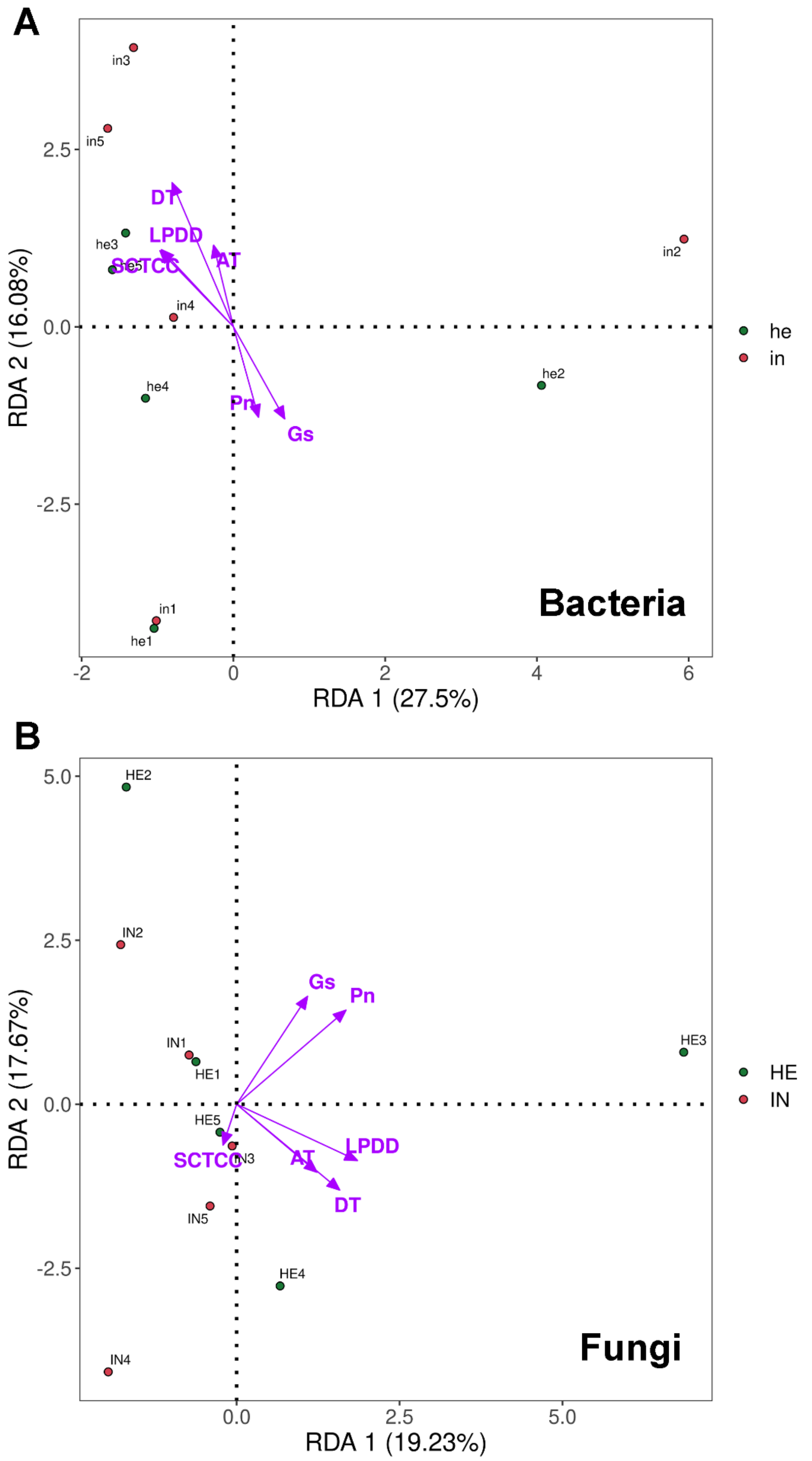

Fig. S4 Redundancy analysis (RDA) of correlation between phyllosphere microbe relative abundance and environment-plant performance model

(A), RDA of phyllosphere bacteria; (B), RDA of phyllosphere fungi.

Table SI Sample name and sampling time

Sampling time	Asymptomatic leaf		Symptomatic leaf		Disease severity
Mid-May (1st sampling)	HE/he1	HE/he1.1	IN/in1	IN/in1.1	0 < DLA ≤ 5% Disease spots sporadically distributed
		HE/he1.2		IN/in1.2	
		HE/he1.3		IN/in1.3	
		HE/he1.4		IN/in1.4	
		HE/he1.5		IN/in1.5	
Mid-June (2nd sampling)	HE/he2	HE/he2.1	IN/in2	IN/in2.1	5% < DLA ≤ 10% Disease area expansion
		HE/he2.2		IN/in2.2	
		HE/he2.3		IN/in2.3	
		HE/he2.4		IN/in2.4	
		HE/he2.5		IN/in2.5	
Early July to late August (3rd sampling)	HE/he3	HE/he3.1	IN/in3	IN/in3.1	10% < DLA ≤ 25% Disease spots begin to connect
		HE/he3.2		IN/in3.2	
		HE/he3.3		IN/in3.3	
		HE/he3.4		IN/in3.4	
		HE/he3.5		IN/in3.5	
Late August to early September (4th sampling)	HE/he4	HE/he4.1	IN/in4	IN/in4.1	25% < DLA ≤ 50% Most of the disease spots are connected and narrow
		HE/he4.2		IN/in4.2	
		HE/he4.3		IN/in4.3	
		HE/he4.4		IN/in4.4	
		HE/he4.5		IN/in4.5	
Early October (5th sampling)	HE/he5	HE/he5.1	IN/in5	IN/in5.1	DLA > 50% Disease spots are all connected and wider
		HE/he5.2		IN/in5.2	
		HE/he5.3		IN/in5.3	
		HE/he5.4		IN/in5.4	
		HE/he5.5		IN/in5.5	

DLA, Diseased Leaf Area, indicates the ratio of diseased area to leaf area.

Table SII Statistical table of 16S rDNA Amplicon Sequencing Alpha diversity index (10 groups)

group	observed_species	shannon	simpson	chao1	ACE	goods_coverage	PD_whole_tree
he1	361	4.484	0.89	432.59	436.253	0.997	30.944
he2	312	4.31	0.873	345.511	356.506	0.998	49.754
he3	186	3.523	0.857	243.559	251.845	0.998	34.596
he4	233	3.28	0.75	302.611	309.73	0.998	26.314
he5	258	3.296	0.741	310.189	325.099	0.998	31.254
in1	360	4.8	0.907	436.857	433.722	0.998	29.23
in2	372	5.386	0.953	403.929	408.42	0.998	34.16
in3	343	5.02	0.921	378.888	389.676	0.998	41.664
in4	280	3.806	0.822	362.632	371.297	0.998	31.461
in5	425	4.71	0.869	471.456	492.675	0.997	49.677

Table SIII Relative abundance by bacterial group analysis of the top35 at the genus level

Taxonomy	he1	he2	he3	he4	he5	in1	in2	in3	in4	in5
Pseudomonas	0.081693	0.014788	0.001209	0.053738	0.010773	0.14406	0.111856	0.287486	0.618044	0.260704
unidentified_Oxyphotobacteria	0.201987	0.277755	0.107571	0.485277	0.490436	0.110336	0.061494	0.16232	0.12999	0.157676
Sphingomonas	0.268915	0.223107	0.32516	0.066479	0.057634	0.329194	0.1447	0.152074	0.052427	0.131145
Phyllobacterium	0.002298	0.001077	0.200162	0.001969	0.000144	0.000946	0.00073	0.000539	0.000461	0.000317
Pantoea	0.067945	0.005733	0.000443	0.007792	0.005686	0.039015	0.005105	0.022251	0.044431	0.004046
Hymenobacter	0.023466	0.064959	0.000263	0.002831	0.029721	0.02124	0.191442	0.037172	0.002759	0.051511
Aureimonas	0.029828	0.063894	0.067179	0.099413	0.154946	0.0244	0.027799	0.020528	0.0189	0.104632
Brevundimonas	0.000114	0.023095	0.100132	0.000317	0.00003	0.000353	0.026656	0.000886	0.000156	0.00006
Methylobacterium	0.013585	0.047926	0.071351	0.123808	0.102621	0.01826	0.069238	0.032892	0.024675	0.053767
unidentified_Enterobacteriaceae	0.000197	0.029834	0.000066	0.00486	0.001113	0.001239	0.012335	0.004357	0.000275	0.007732
unidentified_Rhizobiaceae	0.004309	0.027171	0.010868	0.001406	0.000509	0.004213	0.012119	0.022347	0.007876	0.006458
unidentified_Clostridiales	0.004219	0.023012	0.000263	0.000563	0.000814	0.003375	0.057963	0.000227	0.000078	0.003357
Variovorax	0.006853	0.011916	0.002927	0.000826	0.001712	0.008355	0.050344	0.015315	0.002328	0.015369
Spirosoma	0.00015	0.00061	0.000263	0.002023	0.001083	0.000126	0.001927	0.019792	0.004207	0.02908
Serratia	0.010234	0.001454	0	0.000485	0.000018	0.001406	0.003124	0.000203	0.000126	0.000162
Delftia	0.000018	0.000048	0.036142	0.000084	0.000012	0.00006	0.00015	0.00015	0.000054	0.000048
Stenotrophomonas	0.000341	0.0007	0.000156	0.000192	0.000497	0.000317	0.00076	0.011766	0.001712	0.000598
Buchnera	0.02522	0.020504	0.000006	0.002023	0.008331	0.014998	0.022305	0.000455	0.000317	0.004375
Massilia	0.014579	0.001837	0.000018	0.000108	0.000269	0.021198	0.009169	0.001658	0.000078	0.001556
unidentified_Burkholderiaceae	0.000042	0.003016	0	0.000006	0.00003	0.000162	0.007906	0	0.000054	0.000012
Falsirhodobacter	0.005602	0.000808	0	0.000012	0.000006	0.009636	0.000126	0.000066	0.000012	0.000024
Erwinia	0.008463	0.001466	0.000096	0.001742	0.000036	0.00726	0.00167	0.003752	0.004943	0.000257

Xanthomonas	0.001448	0.000006	0.000054	0.000383	0.000485	0.000778	0	0.015656	0.008439	0.004441
Paenibacillus	0.000467	0.005297	0	0.000006	0	0.000928	0.006523	0.001658	0.000024	0
Frigoribacterium	0.001245	0.000844	0.000305	0.000066	0.000371	0.007158	0.004118	0.000449	0.000694	0.004201
Pelomonas	0	0.000197	0.0072	0	0	0	0.000293	0.000006	0	0
Acinetobacter	0.000162	0.000904	0.000048	0.001831	0.000072	0.00009	0.000353	0.003537	0.000269	0.000054
Novosphingobium	0.000203	0.00079	0.000108	0.000359	0.000676	0.000168	0.001921	0.008044	0.00328	0.007744
unidentified_Lachnospiraceae	0.007116	0	0.000024	0.000473	0.001622	0.009061	0	0.000108	0.000287	0.002783
Enterococcus	0.000491	0.000054	0	0.00237	0.000006	0.00088	0.000203	0.000126	0.00003	0.000012
Rahnella	0.00298	0.00009	0	0.000156	0.000048	0.000682	0.000054	0	0.00003	0.000042
Pedobacter	0.000192	0.000993	0.000168	0.000197	0.000168	0.000162	0.001035	0.004249	0.004255	0.002962
Exiguobacterium	0.000215	0.000838	0	0	0	0.003316	0.000425	0	0	0
Fibrella	0.000006	0.000048	0	0.000066	0.000102	0.000012	0.000275	0.005332	0.000592	0.003884
Deinococcus	0.000036	0.000718	0.000024	0.000006	0.000072	0.000239	0.006805	0.000533	0.000102	0.003106

1

Table SIV Environmental conditions during the period of sampling

Year	Sampling time	Mean air temperature (℃)	Mean dew point temperature (℃)	Mean sky condition total coverage code	Mean liquid precipitation depth dimension - six hour duration (mm)
2018	Mid-May (1st sampling)	20.7086	14.7571	7.1619	36.5556
	Mid-June (2nd sampling)	22.5029	17.0457	7.181	33.2273
	Early July to late August (3rd sampling)	25.9961	22.2199	6.9636	92.3958
	Late August to early September (4th sampling)	25.6944	20.5694	6.9592	62.4667
	Early October (5th sampling)	20.3971	17.56	77,143	24.4546
